# The clinical measurement, measurement method and experimental condition ontologies: expansion, improvements and new applications

**DOI:** 10.1186/2041-1480-4-26

**Published:** 2013-10-08

**Authors:** Jennifer R Smith, Carissa A Park, Rajni Nigam, Stanley JF Laulederkind, G Thomas Hayman, Shur-Jen Wang, Timothy F Lowry, Victoria Petri, Jeff De Pons, Marek Tutaj, Weisong Liu, Elizabeth A Worthey, Mary Shimoyama, Melinda R Dwinell

**Affiliations:** 1 Human and Molecular Genetics Center, Medical College of Wisconsin, Milwaukee, WI, USA; 2 Department of Animal Science, Iowa State University, Ames, IA, USA; 3 Department of Pediatrics, Medical College of Wisconsin, Milwaukee, WI, USA; 4 Department of Surgery, Medical College of Wisconsin, Milwaukee, WI, USA; 5 Department of Physiology, Medical College of Wisconsin, Milwaukee, WI, USA

## Abstract

**Background:**

The Clinical Measurement Ontology (CMO), Measurement Method Ontology (MMO), and Experimental Condition Ontology (XCO) were originally developed at the Rat Genome Database (RGD) to standardize quantitative rat phenotype data in order to integrate results from multiple studies into the PhenoMiner database and data mining tool. These ontologies provide the framework for presenting what was measured, how it was measured, and under what conditions it was measured.

**Results:**

There has been a continuing expansion of subdomains in each ontology with a parallel 2–3 fold increase in the total number of terms, substantially increasing the size and improving the scope of the ontologies. The proportion of terms with textual definitions has increased from ~60% to over 80% with greater synchronization of format and content throughout the three ontologies. Representation of definition source Uniform Resource Identifiers (URI) has been standardized, including the removal of all non-URI characters, and systematic versioning of all ontology files has been implemented. The continued expansion and success of these ontologies has facilitated the integration of more than 60,000 records into the RGD PhenoMiner database. In addition, new applications of these ontologies, such as annotation of Quantitative Trait Loci (QTL), have been added at the sites actively using them, including RGD and the Animal QTL Database.

**Conclusions:**

The improvements to these three ontologies have been substantial, and development is ongoing. New terms and expansions to the ontologies continue to be added as a result of active curation efforts at RGD and the Animal QTL database. Use of these vocabularies to standardize data representation for quantitative phenotypes and quantitative trait loci across databases for multiple species has demonstrated their utility for integrating diverse data types from multiple sources. These ontologies are freely available for download and use from the NCBO BioPortal website at http://bioportal.bioontology.org/ontologies/1583 (CMO), http://bioportal.bioontology.org/ontologies/1584 (MMO), and http://bioportal.bioontology.org/ontologies/1585 (XCO), or from the RGD ftp site at ftp://rgd.mcw.edu/pub/ontology/.

## Background

Integrating phenotype data from multiple experiments and sources is challenging because of the general lack of standardization in how such data is presented. The Clinical Measurement (CMO), Measurement Method (MMO), and Experimental Condition (XCO) Ontologies were developed at the Rat Genome Database (RGD) [1-3] to meet this challenge [4]. The CMO, MMO, and XCO constitute a suite of ontologies designed to provide detailed descriptions of specific, quantitative phenotype data and the experiments that produced them by indicating (1) what was measured, (2) how it was measured, and (3) under what conditions it was measured. Along with the Rat Strain Ontology, these form the basis of the RGD PhenoMiner tool for mining and visualizing quantitative phenotype data [5].

Because the ontologies were designed to work together, their development was originally, and continues to be, coordinated. They were first used to integrate and standardize high-throughput rat phenotype data from the PhysGen Programs for Genomic Applications (PGA) [6,7] and the National BioResource Project for the Rat in Kyoto, Japan (NBRP) [8], and by the COVER project at Washington University in St. Louis in the integration of human cardiovascular phenotype data [4]. The success of these efforts has prompted further development of these ontologies, resulting in expansions of their size, their scope, and their usage. This paper will present details regarding these improvements to the ontologies and information about applications of the ontologies which have recently been implemented.

## Results and discussion

### Increases in the size and scope of the clinical measurement ontology

As stated when this ontology was originally released, the Clinical Measurement Ontology was “…primarily organized on the highest level according to the body system in which the measurement is made” [4]. This is still the case. However, both the size and the scope of the ontology have substantially increased [9]. Between 2012 and 2013, the number of terms has grown from a total of 523 to 1691, the maximum depth of the ontology has now increased to 11, the percentage of classes with a single subclass is 15.6%, and the average branching factor is 0.86 (Table 1). This table also shows that the percentage of classes with two or more parents has increased to 15.8%. Although it is a common practice to limit the number of asserted parents to a single one for each ontology term, the applications for which these ontologies were designed are largely geared toward physiological and clinical researchers. As such and in keeping with our decision from the beginning to use a pragmatic approach to the design of these ontologies, in cases where it seems clear that a researcher would expect to find a relationship between terms it is our practice to assert that parentage. Also, as will be discussed later, we have found that some groups have begun to extract only a small subset of terms from one of these ontologies, to use according to their needs. This practice is facilitated by the assertion of parentage rather than limiting those assertions to a single parent and trusting semantic reasoners to supply the missing relationships. For these applications, artificially limiting the assertions of parentage or conforming to a formalized ontology design pattern (ODP) or structured upper level ontology such as the Basic Formal Ontology (BFO), while perhaps improving the ontology’s logical structure, renders the ontology opaque to many researchers’ attempts to browse the vocabulary to find the term(s) they need. In this respect, as Lord and Stevens commented, “…while realist principles may enable straight-forward modelling for some topics, there are crucial aspects of science and the phenomena it studies that do not fit into this approach; realism appears to be over-simplistic which, perversely, results in overly complex ontological models” [10]. The structures of the ontologies are therefore based on their contents and the established organizational hierarchies understood by the research communities that are using the vocabularies.

**Table 1:** Comparison of ontology statistics between 2012 and 2013

	**Clinical measurement**		**Measurement method**		**Experimental condition**	
	**2012**	**2013**	**2012**	**2013**	**2012**	**2013**
**Total # terms**	523	1691	195	402	110	346
**Defined terms**	328	1427	116	326	76	320
**Percent defined**	62%	84%	59%	81%	69%	92%
**Maximum depth**	7	11	6	8	5	8
**% terms with 2 or more parents**	2.0%	15.8%	<1%	7.2%	1.0%	14.2%
**% terms with single subclass**	10.3%	15.6%	7.7%	14.9%	10.9%	17.1%
**Average branching factor**	0.98	0.86	1.00	0.93	0.99	0.87

Table 1 displays the total number of terms for each ontology as well as the number and percentage of those terms with textual definitions, at the time of the original publication in May 2012 and as of July/August 2013. In addition, basic statistics such as the maximum depth of each ontology and information about the degree of branching for each are included.

**Figure 1:**
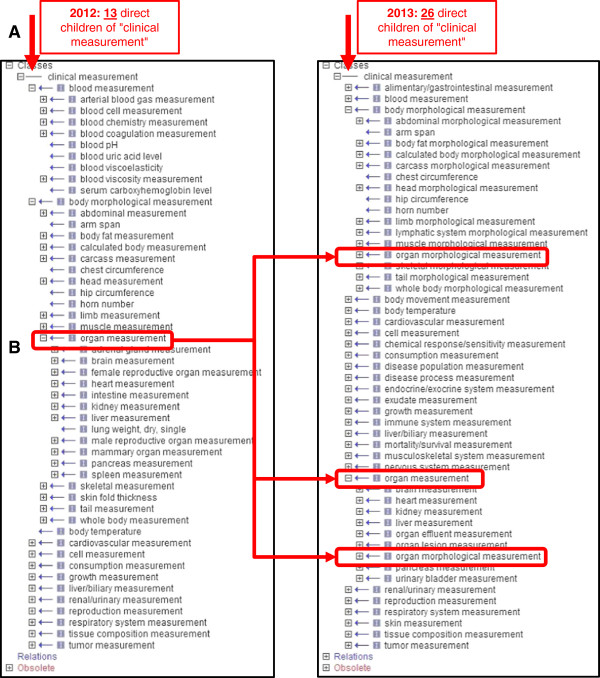
**The clinical measurement ontology 2012 vs. 2013. A.** Additions and improvements to the CMO have resulted in an expansion of both the number of terms and the scope of the ontology. In May of 2012, there were 13 direct child terms under the root “clinical measurement”. As of July 2013, this had increased to 26. The vertical arrows point to the level in the display which corresponds to the vocabulary nodes directly under the root. **B.** Adjustments to the branch for “body morphological measurement” and addition of a new branch for “organ measurement” clarified the morphological terms and allowed for addition of organ-specific physiological terms.

As shown in Table 2 and Figure 1A, the scope has expanded from 13 direct subclasses of the term “clinical measurement” to 26. The new branches include coverage for additional organ systems (alimentary/gastrointestinal system, endocrine/exocrine system, immune system, musculoskeletal system, nervous system, and skin) as well as coverage for measurements that do not necessarily come to mind as “phenotypes”. These include branches related to disease population measurements such as incidence and prevalence, to disease processes such as onset/diagnosis and progression, and to mortality and survival. In these cases, quantitative values are commonly assessed and reported in the literature. For example, researchers will give a number for the percentage of a study population that develop a disease within a given period of time, report the age at which a disease state is detected, or track the proportion of animals in a study population which are surviving at a series of time points.

**Table 2:** Expansion of the scope of the clinical measurement ontology

	**Original branches of the CMO:**	**New branches added to the CMO:**
**Organ systems:**		
	Cardiovascular system	Alimentary/gastrointestinal system
	Liver/biliary system	Endocrine/exocrine system
	Renal/urinary system	Musculoskeletal system
	Reproduction	Nervous system
	Respiratory system	Skin
		Immune system
**Other branches:**		
	Blood measurement	Body movement measurement
	Body morphological measurement	Chemical response/sensitivity measurement
	Body temperature	Disease population measurement (incidence/prevalence)
	Cell measurement	Disease process measurement (onset/diagnosis, progression, severity)
	Consumption measurement	Exudate measurement
	Growth measurement	Mortality/survival measurement
	Tissue composition measurement	Organ measurement
	Tumor measurement	

Table 2 lists all of the direct children of the parent CMO term “clinical measurement” as of July 2013, v2.5. These are divided into the list of terms that existed at the time of the original publication of the ontology on the left and those which have subsequently been added to the ontology on the right. Increases in the scope of the CMO have almost doubled the number of direct subclasses of the parent.

A branch for chemical response and sensitivity measurements which covers the results of a variety of both *in vivo* and *ex vivo* drug and chemical tests was added, as was a branch for “exudate measurements” for use with measurements made on extravasated fluid or other substances.

The term originally labeled as “organ measurement” (CMO:0000068) was changed to “organ morphological measurement” in keeping with its placement under “body morphological measurement” and above terms which described only organ morphology. A new term for “organ measurement” (CMO:0000669) was created directly under the root term “clinical measurement” and linked as a parent to “organ morphological measurement” (CMO:0000068) (Figure 1B). These changes have allowed inclusion of physiological measurements related to the specified organs in addition to their corresponding morphological measurements.

A new branch for “body movement measurement” addresses a common area of study in rodent research that was not covered in the earlier version of the CMO. This branch is designed to include both involuntary movements, such as measurement of the acoustic startle response (CMO:0001519), and voluntary movements. In the rodent research literature, measurements of voluntary movements such as locomotor behavior in an open field apparatus, rearing, or freezing are often presented as measurements of the emotional state of the animal (e.g., anxiety [11]). Although this is a common interpretation of the results, the psychological state is not the actual quantity being measured. Additionally, such movement measurements can be used in other contexts. A cursory search of the rat literature resulted in articles in which movement in an open field apparatus was used to assess learning/memory [12], ethanol-related hyperactivity [13], the sedative effects of drug treatments [14], the locomotor effects of vestibular dysfunction [15], and the effects of cholinergic denervation of the hippocampus [16]. This being the case, the branch was developed as representing measurements of movement in general, not of psychology or emotionality. Also, because the same measurements are made across a number of different types of apparatus, the specifics of the apparatus are assigned via the MMO rather than being included in the CMO terms.

Collaboration with the Animal QTL Database (QTLdb) [17,18] has led to the addition of a substantial number of CMO terms related to agricultural animal assessments. These include terms for measurements commonly used by the agricultural community to assess the composition and yield of milk for cattle and sheep, as well as measurements of fowl eggs, of fat and muscle morphology and fat composition in cattle and pigs, and of feed intake and weight gain in cattle, pigs, sheep, and chickens.

### Increases in the size and scope of the measurement method ontology

**Figure 2:**
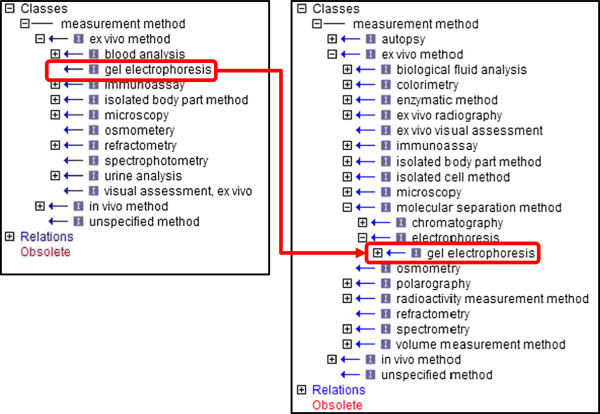
**The measurement method ontology 2012 vs. 2013.** Addition of new terms such as “chromatography” necessitated the creation of a “molecular separation method” branch under “ex vivo method”. The term “gel electrophoresis”, as a type of molecular separation method, was moved from being a direct child of “ex vivo method” into the new branch.

The Measurement Method Ontology covers the domain of the specific methods used to make the measurements represented by the CMO terms, i.e., “how it was measured”. This being the case, development of this ontology is closely coordinated with the development of the CMO and it has likewise increased in size and scope [19]. The number of terms in the MMO has increased from 195 to 402, the maximum depth of the ontology has increased to 8, the percentage of classes with two or more parents has risen to 7.2%, the percentage of classes with a single subclass is 14.9%, and the average branching factor is 0.93 (Table 1). The MMO is subdivided into two major branches: “in vivo method” for methods performed in or on a living body, and “ex vivo method” for procedures performed outside the living body. Improvements to the *in vivo* branch include the addition of terms for body movement methods, such as subbranches for types of test enclosures, mazes, and treadmills, and addition of more general branches for “flowmetry” and “body fluid collection method”. New subbranches under “ex vivo method” include radioactivity and volume measurement methods, as well as a branch for “isolated cell method” which corresponds to expansion of the CMO “cell measurement” branch. In several cases, what was originally a single term has been expanded into a larger branch. For instance “gel electrophoresis”, originally a direct child of “ex vivo method”, now appears within the “molecular separation method” branch of the MMO (Figure 2).

### Increases in the size and scope of the experimental condition ontology

Because incorporation of data from new areas of research requires the addition of new condition terms, the Experimental Condition Ontology has expanded from 110 to 346 terms, the maximum depth of the ontology has now increased to 8, the percentage of classes with two or more parents is 14.2%, the percentage of classes with a single subclass is 17.1% and the average branching factor is 0.87 (Table 1) [20]. New branches under “experimental condition” include “controlled visible light condition”, “controlled in situ organ condition” and “pathogen”. New terms include “sample resting period”, which was necessitated by experiments in which separate measurements were made on a sample before and after the sample was allowed to sit for a specified period of time. The only difference in the conditions between the two values was the “sample resting period”. In addition, a term for “perfusate” was added within the more general “solution” branch to describe experiments performed on isolated organs. The terms “surgical implantation” and “surgical removal” were moved under the new “surgical manipulation” term, and “fasting” was incorporated into the existing “diet” branch.

The most extensive additions to the XCO were made in the existing “chemical” branch. Originally, the branch included four subclasses: “anesthetic”, “neoplasm inducing agent”, “polycyclic arene”, and “steroid”. As the number of subclasses increased (at one point reaching 25 direct children of “chemical”) it became clear that a better organizational strategy was needed. Table 3 compares the original children of “chemical” with the current structure of the branch. Following the lead of the Chemical Entities of Biological Interest (ChEBI) ontology [21,22], the branch was split into two major subbranches: “chemical with specified function” and “chemical with specified structure”. Classes representing functional roles such as “disease inducing chemical” and “neurotransmitter” have been moved under the former term. Those representing structural classifications such as “hydrocarbon” and “sulfonamide” are now found under the latter. This structure facilitates browsing for researchers. In this way, whether a chemist is looking for a nitrosourea or a physiologist is looking for a mutation inducing agent both will find the commonly used mutagen N-ethyl-N-nitrosourea (ENU) where they would intuitively expect it to be. Note that the term “polycyclic arene”, previously a direct child of “chemical”, has been moved to the appropriate level within the more general “hydrocarbon” subbranch. Where possible, the corresponding ChEBI ID is given as a cross-reference for the term in the XCO.

**Table 3:** Expansion of the “chemical” branch of the experimental condition ontology

**Original subclasses of “chemical” in the XCO**	**Current organization of terms under “chemical” in the XCO**	
Anesthetic	**Chemical with specified function**	
Neoplasm inducing agent	Activator	
Polycyclic arene	Anesthetic	
Steroid	Antigen	
	Antioxidant	
	Buffer	
	Disease inducing chemical	
	Diuretic	
	Enzyme substrate	
	Hormone	
	Indicator	
	Inhibitor	
	Mutation inducing agent	
	Neoplasm inducing agent	
	Neurotransmitter	
	Toxic substance	
	Vasoactive chemical	
	**Chemical with specified structure**	
	Alcohol	
	Amino acid	
	Carbohydrate	
	Chemical nanoparticle	
	Hydrocarbon	
	Ion/salt	
	Labeled chemical	
	Nitrosourea	
	Nucleic acid	
	Peptide/protein	
	Steroid	
	Sulfonamide	

Table 3 highlights the expansion and reorganization of the XCO branch under the subclass “chemical”. The original four subclasses are shown on the left. As of August 2013, v3.0, the branch has been divided into two major subcategories: “chemical with specified function” and “chemical with specified structure” and the terms which were previously direct children of “chemical” have been moved under one of these two. In addition to more closely following the familiar structure of the ChEBI ontology, the new organization facilitates browsing.

Consideration was given to simply using the ChEBI ontology for chemical conditions. However, ChEBI is fundamentally an ontology of chemical structures. We would argue that the concept of the use of a chemical as an experimental condition is qualitatively different than that of a chemical as a structure or molecule. In addition, ChEBI is often used in annotation of molecular level gene-chemical interactions which differs from an annotation of a chemical bolus or solution being administered as an experimental stressor. The decision was therefore made to include terms for chemical conditions in the XCO and express the relationship between such a condition and the structure and role of the referenced chemical via cross references to the appropriate ChEBI ID.

### Improvements to textual definitions

Work is currently ongoing to both increase the proportion of terms with textual definitions and standardize the format of those definitions for all three ontologies. As shown in Table 1, at the time of the original publication 62% (328/523) of CMO, 59% (116/195) of MMO, and 69% (76/110) of XCO terms had assigned definitions. This proportion has increased to 84% (1427/1691), 81% (326/402), and 92% (320/346), respectively.

As terms are defined, definitions for words or phrases that will be reused are added to a growing list of standardized definition “fragments”. Definitions are currently written manually rather than being automatically generated, but the structure is based on the standard genus-differentia model so that the definition of the child includes the definition of the parent with the addition of applicable differentiating information. As much as possible, each definition is written in such a way that it “stands alone”, that is, so that the user is not required to go up the tree to find the definition of the more general concept. In this way, the definition of “plasma glucose level” (CMO:0000042) has been expanded from “*The level of glucose found in a specific volume of plasma*” to “*Measurement of the amount of glucose, the monosaccharide sugar, C6H12O6, occurring widely in plant and animal tissues which is one of the three dietary monosaccharides that are absorbed directly into the bloodstream during digestion, is the end product of carbohydrate metabolism, and is the chief source of energy for living organisms, in a specified volume of plasma, the fibrinogen-containing fluid portion of the blood in which the particulate components are suspended*” in order to incorporate the fragments which define level, glucose, and plasma, respectively. A list of the standard definition fragments currently in use is provided as Additional file 1.

### Additional improvements

Additional improvements have been implemented to bring the development of the three ontologies into line with established best practices [23]. Because the development of these ontologies was carried out collaboratively, over time textual information such as definition source Uniform Resource Identifiers (URI) was entered using a variety of formats. For instance, at one point “Dorland’s Illustrated Medical Dictionary, 31st Edition” [24], one of a number of sources used frequently for all three ontologies, was represented by 14 slightly different URIs, most of which differed by as little as the inclusion of a period or apostrophe, or the designation of the edition. Although such differences are simple for the human mind to interpret, they make the information difficult to interpret by parsers and other computer applications. These have all been standardized to “Dorland:Dorlands_Illustrated_Medical_Dictionary--31st_Ed”. As this example also illustrates, definition source URIs have been reformatted to remove all “non-URI” characters as defined by the World Wide Web Consortium (W3C) [25]. According to the W3C document, the characters permitted for a URI which do not have a reserved purpose include upper- and lowercase letters (A-Z/a-z), digits (0–9), hyphen, period, underscore, and tilde. All definition source URIs for the CMO, MMO, and XCO have been reformatted so that only those characters are used. Also, to further increase the standardization and traceability of definitions, the applicable ISBN number has been added to the list of source URIs when a hard-copy book is used rather than an online resource. A representative list of definition source URIs has been provided as Additional file 2.

Finally, a standardized system for file versioning has been implemented, including minor version increments for ongoing term and definition additions and major version increments for global changes to the contents or structure of the ontologies. For example, the standardization of the definition source URIs was considered a global change to the contents of the ontologies and warranted the increase of the major version number for each ontology from 1.x to 2.0. The current version number for each ontology can be found as the “data-version” notation in the header of the ontology file (See Additional file 3). The data version for each file is also given in the list of ontology files available on the applicable NCBO BioPortal ontology page. The version numbers referenced in this paper are v2.5 for the CMO, v2.3 for the MMO, and v3.0 for the XCO.

### Expanded applications

Successful use of the three ontologies for their original intent has spurred expansion into new areas. The Animal QTLdb has instituted the use of the CMO, mapping existing QTL trait descriptions to measurement terms. The CMO term is used alone or in conjunction with the Vertebrate Trait Ontology (VT) [26] and/or the Product Trait Ontology [27] to cover the various concepts represented by the original Animal Trait Ontology (ATO) [28]. Currently, over 600 ATO traits have been mapped to 267 unique CMO terms, and a total of 9077 animal QTL and SNP association data have been annotated using the CMO (Table 4).

**Table 4:** QTLs annotated with CMO terms at the animal QTLdb

**Species**	**# QTLs annotated with CMO terms**	**Number of QTLs, by species**	**Percentage of QTLs annotated with CMO**
**Cattle**	3431	7117	48.2%
**Pig**	2933	8402	34.9%
**Chicken**	2315	3808	60.8%
**Sheep**	320	789	40.6%
**Rainbow trout**	78	127	61.4%
**Total**	9077	20243	44.8%

Table 4 lists the number by species and the total number of QTLs which have been associated with any term from the Clinical Measurement Ontology at the Animal QTLdb as of July 2013.

**Figure 3:**
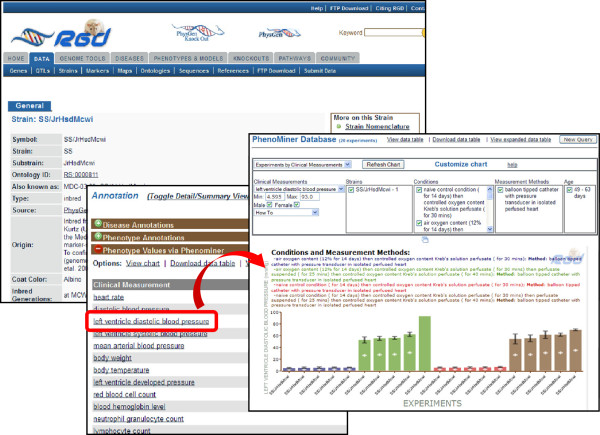
**Access to strain-specific quantitative phenotype data from RGD strain report pages.** All available quantitative phenotype data for a strain is accessible from the RGD strain report page’s phenotype profile. In the section labeled “Phenotype Values via PhenoMiner”, select a CMO term to view values for that strain.

RGD has substantially expanded its use of the CMO, MMO, and XCO for integration of complex datasets in the PhenoMiner project. The number of averaged or summary records in the PhenoMiner database has increased from approximately 13,000 in May, 2012 to now over 69,000. This includes incorporation of additional high-throughput data from the PGA and the PhysGen Knockout project for the rat [29] as well as records derived from manual review of the literature to find and integrate quantitative phenotype data. Such data was previously difficult to locate, because it is often dispersed in tables, text, figures, and figure legends incorporated in the body of the paper or included with the supplementary data, and was even more difficult to compare across studies. Easy access to consolidated results across rat strains and experiments is now available in the RGD PhenoMiner tool [30]. Results for a single strain can be accessed directly in PhenoMiner or from the strain phenotype profile section, labelled “Phenotype Values via PhenoMiner”, on the RGD strain report page. All CMO terms for which data exists in the PhenoMiner database are listed and terms link to the corresponding data in the tool display (Figure 3).

High-throughput phenotyping projects such as the PGA often store the quantitative data from each individual rat that is tested. When such data is available each individual measurement is stored separately in the PhenoMiner database, in addition to being grouped and averaged to form a subset of the aforementioned summary records. Currently the number of individual records is over 563,000.

**Figure 4:**
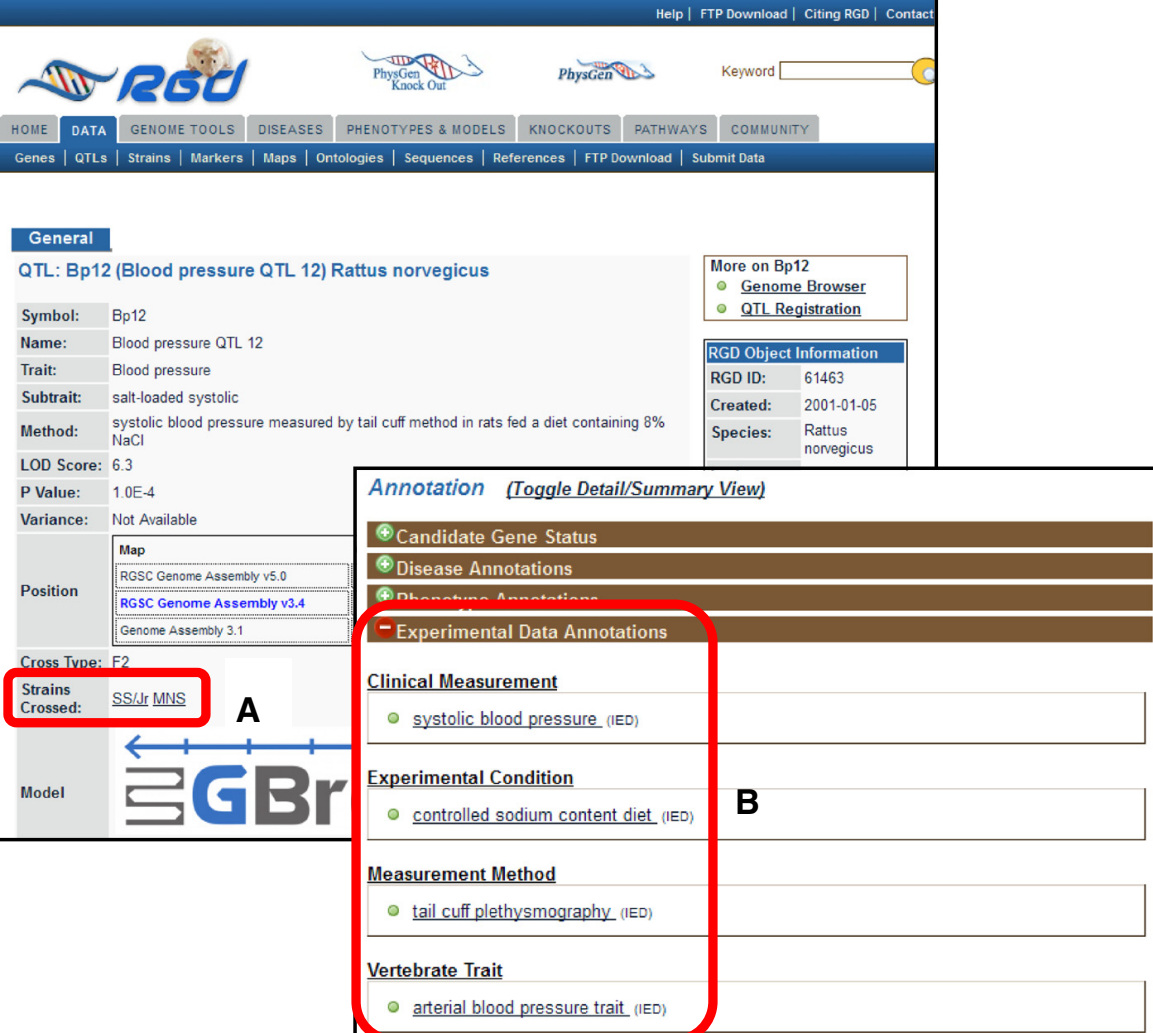
**Use of the CMO, MMO, XCO, VT, and RS ontologies to annotate RGD QTLs.** The RS Ontology **(A)** and the CMO, MMO, XCO, and VT Ontologies **(B)** are used at the Rat Genome Database to standardize the presentation of the rat strains crossed, the specific measurement that was made, the method that was used to make that measurement, the conditions under which the measurement was made, and the specific trait that was measured, respectively. Annotations are assigned an evidence code of “IED” or “inferred from experimental data” to indicate the type of evidence (i.e., experimental) which supports the use of these terms.

CMO, MMO, XCO, VT, and Rat Strain (RS) ontology terms are also assigned to QTL records at RGD (Figure 4) with over 80% (1578/1911) of RGD’s rat QTLs annotated to date. This provides clear experimental design information to users, allows the user to examine, query, and group data by experimental parameters, and links specific sites on the rat genome to the quantitative measurement data in PhenoMiner.

In addition to extraction of phenotype data from the literature by curators, researchers who carry out phenotyping projects on rat strains are encouraged to submit their data directly to RGD. A submission form has been posted on the RGD website to facilitate the process [31]. Researchers collaborate with RGD staff members during the submission process to ensure that their data is integrated into the resource correctly and in a timely fashion.

Researchers are also encouraged to submit term requests for inclusion in the ontologies and/or to suggest modifications and improvements to the vocabularies. Those wishing to submit such requests and suggestions can do so using the “Contact Us” link at the top of the RGD webpages [1] or the contact information supplied on the appropriate BioPortal ontology description pages [32]. Plans are underway to implement tracking software, such as a SourceForge web page [33], to facilitate this process.

Beyond RGD and the Animal QTLdb, the CMO, MMO, and XCO ontologies and the associated QTL annotation data are being used by researchers and other databases via the freely-accessible RGD ftp site. In the past six months, each of the ontologies has been downloaded from the site between 190 and 314 times (CMO: 314 requests; MMO: 211 requests; XCO: 190 requests). The total number of downloads of the data annotation files were 71 for the CMO, 68 for the MMO, and 67 for the XCO. Analysis of the ftp logs shows that the file requests originate from a variety of institutions including universities, medical schools, government institutes and pharmaceutical companies, and from locations in the United States, Europe and Asia, demonstrating the utility of both the ontologies themselves and the associated annotations.

Although these ontologies were originally designed to be used together, they also have utility individually. One such example was recently demonstrated at the 4th International Conference on Biomedical Ontology where Goldfain *et al.* presented their work on the use of ontologies to contextualize the measurement of vital signs in individuals [34]. They use a subset of the XCO to incorporate conditions such as “standing position” (XCO:0000083).

For researchers interested in using the ontologies or the associated data, or in submitting their own data for incorporation into the data set, help is available on the RGD website. Recently updated help pages [35] give information on ontologies in general and their use at RGD, as well as detailed instructions on the use of the PhenoMiner tool, the Phenotypes and Models portal, and the QTL report pages. Tutorial videos such as the “Introduction to the RGD Phenotypes and Models Portal” video [36] demonstrate step-by-step the use of specific tools. The RGD “Introduction to Biomedical Ontologies” tutorial series [37] is geared toward the “ontology novice” and gives basic information about what an ontology is and how it might be used. Finally, help is always available by contacting the RGD curators and developers via the “Contact Us” link at the top and bottom of any RGD web page [1].

### Future directions

There are continuing efforts to standardize both the structure and content of the ontologies. Future development efforts will include the use of standard ontology tools and semantic reasoners, as well as continuing consultations with domain experts to more systematically fill in gaps. Also, in the early stages of development, a number of instrumentation terms were included as measurement methods, for example, “oral thermometer, digital” (MMO:0000196) [19]. However, this term for an object was an is_a subclass of “thermometry”, a method, creating an obvious problem since an object is not a method. Work is underway to review the ontologies both manually and through the use of reasoners to find and correct these types of logical inconsistencies.

Efforts are also underway to add systematic cross referencing from CMO, MMO and XCO terms to related concepts in other ontologies. As previously mentioned, cross references from XCO terms to ChEBI have already been added. Going forward, we anticipate adding similar cross references to ontologies related to disease, cell types, and anatomy. Results from NCBO’s Mappings tool [38,39] will be used as a starting point for finding and documenting such inter-ontology relationships. In addition, we are investigating algorithms which may enable us to map between related terms even when the terms do not use identical text.

In keeping with the common practice for ontology development, the terms, class definitions and structure of these ontologies will be reviewed yearly or as needed to ensure that they remain up to date with advances in the associated research domains and that they conform to both initial and newly identified development requirements [40]. As the number of collaborating domain experts for the development of these ontologies grows, regular discussions with those collaborators will be scheduled to review terms, ontology structure and definitions. Our location at the Medical College of Wisconsin is ideal in this respect since MCW houses a large and varied community of basic rat researchers, clinical researchers and clinicians. Such collaborations have already helped us improve the ontologies.

Finally, in order to make these ontologies usable for tools and software designed for OWL-formatted vocabularies, we will make the CMO, MMO and XCO available in the OWL format in the near future.

## Conclusions

Development of the three ontologies has been essential for the integration of complex phenotype data at RGD. Annotations derived from both high-throughput data and a wide variety of literature-derived QTL data have been incorporated using these ontologies. Increases in the scope of the data being curated through inclusion of studies from diverse areas of research have necessitated substantial increases in the size and scope of all three ontologies. The data used for the original development of these ontologies was heavily weighted toward cardiovascular traits and related phenotypes. High-throughput data from a subset of the Program for Genomic Applications data (PGA) [6,7] and standard phenotypes from the National BioResource Project for the Rat in Kyoto, Japan [8] steered development of the ontologies in this direction. More recently, incorporation of data from QTL studies as diverse as alcohol intake, cancer susceptibility, limb length, joint inflammation, and movement and behavior, as well as collaboration with the Animal QTLdb, have prompted major expansion of the ontologies [5].

Recent advances in both the ontologies themselves and their application have demonstrated the utility of these vocabularies for facilitating the incorporation of data from diverse sources. The use of multiple ontologies to describe individual data types across multiple studies serves to integrate the data while maintaining the aspects that are unique to each study or each measurement. This has been demonstrated by RGD’s PhenoMiner data and by annotation of QTL records at RGD and the Animal QTLdb. Measurements, methods and/or conditions are often shared across studies and even across species. For instance, blood chemistry measurements such as blood cholesterol level, blood glucose level, and hematocrit are available for species from human to chicken. The use of ontologies such as the CMO allows querying of records for multiple species across multiple databases. This cross-species use of shared ontologies gives researchers the ability to access data that previously might have been considered unrelated but is now revealed to be both related and important to consider.

“Ontology development is necessarily an iterative process”, as one tutorial on ontology development put it [41]. This paper describes the most recent iteration of the development process for the Clinical Measurement, Measurement Method, and Experimental Condition Ontologies. As the development process continues, new concepts are continually being added and application of these ontologies is continually expanding, resulting in a greater ability to integrate, consolidate, and compare phenotypic measurement data from diverse sources.

## Methods

The Clinical Measurement Ontology, Measurement Method Ontology, and Experimental Condition Ontology are being developed using the Open Biomedical Ontology (OBO) format. The OBO-Edit software [42] is utilized to add, move, merge, and delete terms as needed. This tool also provides quality control for violations of the accepted best practices for ontology development. Such checking is utilized to find and correct such violations.

The need for new terms is established through a collaborative process within and between the groups at RGD and Animal QTLdb. As curation of new and existing research articles proceeds, the existing vocabularies are examined before a new term request is made. If none of the existing terms is deemed appropriate for use, a request is logged for one or more new terms. Term requests are further reviewed by the ontology developer to ensure the format and wording of the putative new term agrees with pre-existing standards. Literature searches, general internet searches, and consultations with domain experts are utilized to establish the proper placement of new terms and the construction of both standardized and individual term definitions.

Ontology files are exported from OBO-Edit and uploaded to the NCBO BioPortal site [32,43-45] and RGD’s ftp site [46] as needed. During the upload process, version numbers are incremented and the new version numbers added to the file headers.

These ontologies are freely available for download and use from the NCBO BioPortal website at http://bioportal.bioontology.org/ontologies/1583 (CMO), http://bioportal.bioontology.org/ontologies/1584 (MMO), and http://bioportal.bioontology.org/ontologies/1585 (XCO), or from the RGD ftp site at ftp://rgd.mcw.edu/pub/ontology/.

## Competing interests

The authors declared that they have no competing interests.

## Authors’ contributions

JRS had primary responsibility for recent development of the ontologies, participated in application of the ontologies, and drafted the manuscript. CAP contributed to ontology development and use of the CMO at the Animal QTLdb and reviewed the manuscript. RN participated in development and application of the ontologies. SJFL contributed to ontology development, oversaw the applications of the ontologies at RGD, and reviewed the manuscript. GTH contributed term suggestions, participated in the application of the ontologies, and reviewed the manuscript. SJW contributed term suggestions, participated in the application of the ontologies, and reviewed the manuscript. TFL and VP participated in the application of the ontologies. JDP supervised website and database development. MT participated in maintaining the RGD website and database and assisted in loading ontologies. WL had primary responsibility for loading ontologies and participated in development and maintenance of the PhenoMiner database and webpages. EAW co-supervised RGD projects. MS conceived of the study, created the ontologies, supervised RGD projects, contributed to ontology development and design of the PhenoMiner website, and reviewed the manuscript. MRD had primary responsibility for the PhenoMiner project, contributed to ontology development and design of the PhenoMiner website, and reviewed the manuscript. All authors read and approved the final manuscript.

## Supplementary Material

Additional file 1 **Standard definition fragments.** Additional file 1 is a plain text (i.e., .txt file) list of definition fragments which are used to construct standardized definitions for ontology terms. Each entry contains the text of the word or phrase being defined, the definition, and the definition source in the standardized format for source URIs. Click here for file

Additional file 2 **Standard sources for term definitions.** Additional file 2 is a plain text (i.e., .txt file) list of definition sources in the current standardized URI format. Sources include clinical and veterinary texts, standard medical and general dictionaries, websites, and review articles. In addition to these “reusable” sources, research and review articles covering specific details of measurements, methods or conditions are used in some cases. Click here for file

Additional file 3 **Systematic versioning of ontology files.** Additional file 3 is a pdf version of the header of the CMO ontology file. The data-version tag in the ontology file header, or metadata, shows the version number of that file. Minor version number changes, e.g., 2.1 to 2.2, indicate ongoing ontology development such as addition of new terms and definitions. Major version number changes such as 1.x to 2.0 indicate global changes to the ontology or major changes to its structure. Click here for file
