# Supplementary material for: The clinical measurement, measurement method and experimental condition ontologies: expansion, improvements and new applications
Source: J Biomed Semantics. 2013 Oct 8;4:26. doi: 10.1186/2041-1480-4-26 (PMC3882879; doi:10.1186/2041-1480-4-26)
Supplement: Additional file 3 — Systematic versioning of ontology files. Additional file 3 is a pdf version of the header of the CMO ontology file. The data-version tag in the ontology file header, or metadata, shows the version number of that file. Minor version number changes, e.g., 2.1 to 2.2, indicate ongoing ontology development such as addition of new terms and definitions. Major version number changes such as 1.x to 2.0 indicate global changes to the ontology or major changes to its structure. [file 2041-1480-4-26-S3.pdf]

```
format-version: 1.2
date: 19:03:2013 18:58
saved-by: JSmith
auto-generated-by: OBO-Edit 2.3
default-namespace: Clinical_Measurement.ontology
data-version: 2.2

[Term]
id: CMO:0000000
name: clinical measurement
def: "A quantitative or qualitative value which is
```
